# Intracranial arterial stenosis associated with Hashimoto’s disease: angiographic features and clinical outcomes

**DOI:** 10.1186/s12883-020-01923-w

**Published:** 2020-09-14

**Authors:** Eika Hamano, Masaki Nishimura, Hisae Mori, Tetsu Satow, Jun C. Takahashi

**Affiliations:** Department of Neurosurgery, National Cerebral and Cardiovascular Center, Kishibe-Shinmachi 6-1, Suita City, Osaka 564-8565 Japan

**Keywords:** Anti-thyroid antibody, Hashimoto’s disease, Intracranial arterial stenosis, Moyamoya disease

## Abstract

**Background:**

Hashimoto’s disease has reportedly been associated with stroke; however, cerebrovascular morphology and clinical course remain poorly documented. The present study aimed to determine the angiographic features and clinical outcomes of intracranial arterial stenosis (IAS) associated with Hashimoto’s disease in a retrospective cohort.

**Methods:**

Overall, 107 adult patients with IAS were screened for anti-thyroid antibodies; of these, 26 patients tested positive. The 42 affected hemispheres were classified into subgroups according to the steno-occlusion site and the development of abnormal collateral (moyamoya) vessels. These subgroups were dichotomized into moyamoya vessels positive (MM type) and negative (non-MM type). The initial presentation, IAS progression, and vascular events during the follow-up period were compared.

**Results:**

The following sites of stenosis were identified: the bifurcation of the internal carotid artery in 11 (26.2%), M1 or A1 in 29 (69.0%), and more distal (M2-M4/A2-A4) in 2 (4.8%) hemispheres. Further, 17 hemispheres were categorized into the MM type and 25 were classified into the non-MM type. During the follow-up period (mean 2.5 years), IAS progression was identified in 8 (32%) hemispheres of the non-MM type and 0 (0%) hemispheres of the MM type (*p* = 0.041). Ischemic attacks occurred in 5 (20.0%) hemispheres of the non-MM type (4.6%/year) and 0 hemispheres of the MM type (*p* = 0.08). Further, 4 (23.5%) hemispheres of the MM type experienced intracerebral hemorrhage, whereas none of the non-MM type hemorrhaged (*p* = 0.012).

**Conclusions:**

Hashimoto’s disease-associated IAS exhibits various angiographic morphologies, resulting in different clinical presentations. Screening for anti-thyroid antibodies and careful management based on vascular morphology appears important in adults with IAS.

## Background

Hashimoto’s disease is a common autoimmune disease characterized by chronic thyroiditis caused by anti-thyroid antibodies that can lead to hypothyroidism [1, 2]. Several studies have revealed that stroke is more common in patients with Hashimoto’s disease than in the normal population [3–6]. Moreover, a recent study reported that the prevalence of anti-thyroid antibodies is higher in moyamoya disease, which is characterized by bilateral intracranial arterial stenosis (IAS), than in non-moyamoya stroke [7]. Intracranial angiographic examinations have widely been performed for Graves’ disease, another autoimmune thyroid disease that can also induce ischemic stroke [8]. However, the intracranial vascular morphology and clinical course of Hashimoto’s disease have poorly been documented. In the present study, we aimed to identify the angiographic features and clinical outcomes in patients with Hashimoto’s disease-associated IAS.

## Methods

This retrospective study was approved by the ethics board at the authors’ institute (M30–013). Individual patient consent was not required from patients. The opportunity to opt out of the study was always available to the research participants.

### Participants

From January 2014 to July 2019, 390 adults (aged ≥20 years) with IAS were hospitalized for neuroradiological examination. Of these, 107 patients were examined for serum autoimmune antibodies, including anti-thyroid antibodies and thyroid hormone levels. In principle, from 2014 to 2017, patients were selected for serum testing using the following criteria: (1) IAS that was unlikely to have been caused by atherosclerosis and (2) IAS that did not strictly fulfill the clinical and angiographic criteria of moyamoya disease [steno-occlusive lesion involving the terminal portion of the internal carotid arteries (ICAs) with accompanying moyamoya vessels]. We observed that the positive rate of anti-thyroid antibodies was higher than expected in IAS, regardless of their vascular morphology. Accordingly, after January 2018, the criteria were expanded to include patients whose angiographic findings were compatible with moyamoya disease. Diagnosis of Hashimoto’s disease or its suspected condition was established as per the guidelines for the Diagnosis of Chronic Thyroiditis (Hashimoto’s disease) established by the Japan Thyroid Association [9]. Briefly, patients were diagnosed with Hashimoto’s disease when they exhibited both thyroid gland swelling without any other cause (e.g., Graves’ disease) and positive anti-thyroid peroxidase antibodies (TPOAbs) and/or anti-thyroglobulin antibodies (TgAbs). Patients who exhibited positive anti-TPOAb or anti-TgAb with no thyroid goiter were suspected to have Hashimoto’s disease. According to the reference level value of the institute, positive anti-TPOAb and anti-TgAb were defined as ≥5.61 international unit (IU)/mL and ≥ 4.11 IU/mL, respectively.

A total of 30 patients exhibited positive anti-TPOAb or anti-TgAb; of these, 4 who exhibited hyperthyroidism and were thereafter diagnosed with Graves’ disease were excluded. Thus, 26 patients (24.3%) were finally included in this study (definite 1, suspected 26).

### Angiographic classification of IAS (Fig. 1)

All patients underwent digital subtraction (DS) angiography. The affected hemispheres were classified into five groups according to the location of the arterial steno-occlusion and development of abnormal collateral vessels (moyamoya vessels):

- Group A: stenosis/occlusion of the ICA bifurcation with moyamoya vessels
- Group B: stenosis/occlusion of the M1/A1 with moyamoya vessels
- Group C: stenosis/occlusion of the ICA bifurcation without moyamoya vessels
- Group D: stenosis/occlusion of the M1/A1 without moyamoya vessels
- Group E: stenosis/occlusion of the M2-M4/A2-A4 without moyamoya vessels

**Fig. 1:**
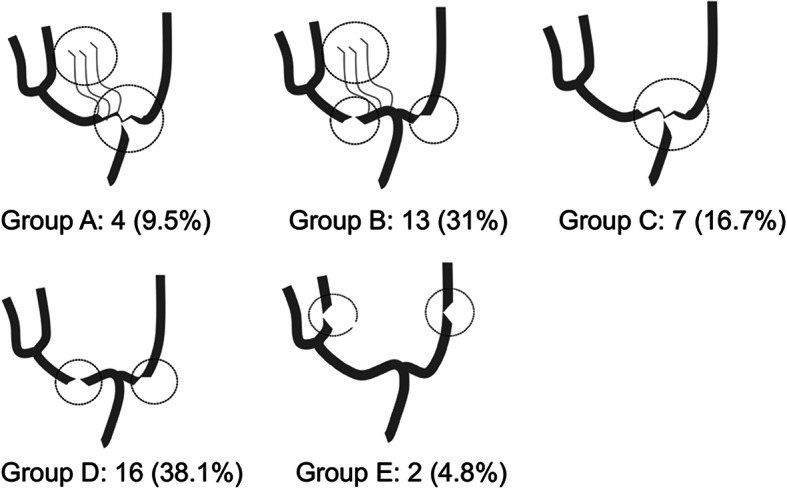
Angiographic classification of the 42 affected hemispheres. Group A: stenosis/occlusion of IC bifurcation with moyamoya vessels. Group B: stenosis/occlusion of M1/A1 with moyamoya vessels. Group C: stenosis/occlusion of IC bifurcation without moyamoya vessels. Group D: stenosis/occlusion of M1/A1 without moyamoya vessels. Group E: stenosis/occlusion of M2–4/A2–4 without moyamoya vessels

All the affected hemispheres exhibited IAS in the anterior circulations. No hemisphere exhibited proximal ICA stenosis with normal ICA bifurcation. No hemisphere exhibited both stenosis/occlusion of the M2-M4/A2-A4 and moyamoya vessels.

The hemispheres in Groups A and B were defined as the moyamoya vessels positive (MM) type, whereas those in Groups C–E were defined as the moyamoya vessels negative (non-MM) type. In the MM type hemispheres, the development of collateral vessels (periventricular anastomosis) was evaluated according to the criteria of the supplemental studies of the Japan Adult Moyamoya Trial (Table 1) [10, 11].

**Table 1:** Definition of positive development of periventricular anastomosis [10, 11]

**Lenticulostriate anastomosis**	▪ Extreme dilation and extension of the lenticulostriate arteries beyond the level of the pericallosal artery
**Thalamic anastomosis**	▪ Extreme dilation and extension of the thalamic perforators beyond the position of the medial posterior choroidal artery
**Choroidal anastomosis**	▪ Extreme dilation and extension of the choroidal artery with sudden deviation from the shape of the lateral ventricle at its peripheral portion to connect to the medullary artery in the anteroposterior view.
	▪ Extreme extension of the anterior choroidal or lateral posterior choroidal artery beyond the atrium of the lateral ventricle to reach the body of the lateral ventricle.
	▪ Connection of the medial posterior choroidal artery to the pericallosal artery by penetrating the corpus callosum.

### Clinical management and follow-up

All the hemispheres were evaluated using single photon emission computed tomography (SPECT) and/or positron emission computed tomography (PET) at the time of diagnosis. Direct bypass surgery [superficial temporal artery (STA)–middle cerebral artery (MCA) anastomosis] was performed within 3 months after diagnosis if the patient exhibited severe hemodynamic failure. All patients were semiannually followed-up using serial magnetic resonance (MR) imaging and monitored for stenosis progression and cerebrovascular events, including transient ischemic attacks (TIAs), intracranial hemorrhage, and cerebral infarction. If MR angiography detected stenosis progression, DS angiography was performed to confirm this finding. The time from diagnosis to these incidents was compared between the MM type and non-MM type hemispheres. This duration was also compared between patients with hypothyroidism (T4 ≤ 1.1 ng/dL and/or TSH ≥ 5.5 μU/mL) and those without hypothyroidism. The modified Rankin scale (mRS) scores of the patients were evaluated at the final follow-up.

### Statistical analyses

A t-test, Mann–Whitney’s U test, chi-squared test, or Fisher’s exact test was used for comparing the baseline characteristics of the two groups. Kaplan–Meier survival analysis with a log-rank test was performed for comparing the length of time without an adverse event (IAS progression, ischemic events, and intracranial hemorrhage) in each group. Two-sided *P* values < 0.05 were considered to indicate statistical significance. All analyses were performed using JMP software, ver. 11 (SAS Institution, Cary, USA).

## Results

### Patient characteristics

Table 2 shows the baseline characteristics of the 26 patients. The mean age ± standard deviation (SD) was 56.4 ± 13.2 years (range 35–79 years). The initial presentation was TIAs in 12 patients, cerebral infarction in 7, and intracranial hemorrhage in 3; 4 patients were asymptomatic.

**Table 2:** Characteristics of 26 IAS patients with positive anti-thyroid antibodies

	No. of patients
Number of patients	26
Mean age at diagnosis ± SD (range)	56.4 ± 13.2 (35–79)
Females	19 (73.1%)
Bilateral	16 (61.5%)
Symptoms at onset	
TIA	12 (46.2%)
Cerebral infarction	7 (26.9%)
Hemorrhage	3 (11.5%)
Asymptomatic	4 (15.4%)
Hypertension	19 (73.1%)
Diabetes mellitus	5 (19.2%)
Hyperlipidemia	10 (38.5%)
Hypothyroidism	16 (61.5%)
Family history	
Moyamoya disease	3 (11.5%)
Thyroid disease	1 (3.8%)

### Angiographic features and clinical presentations at diagnosis

**Fig. 2:**
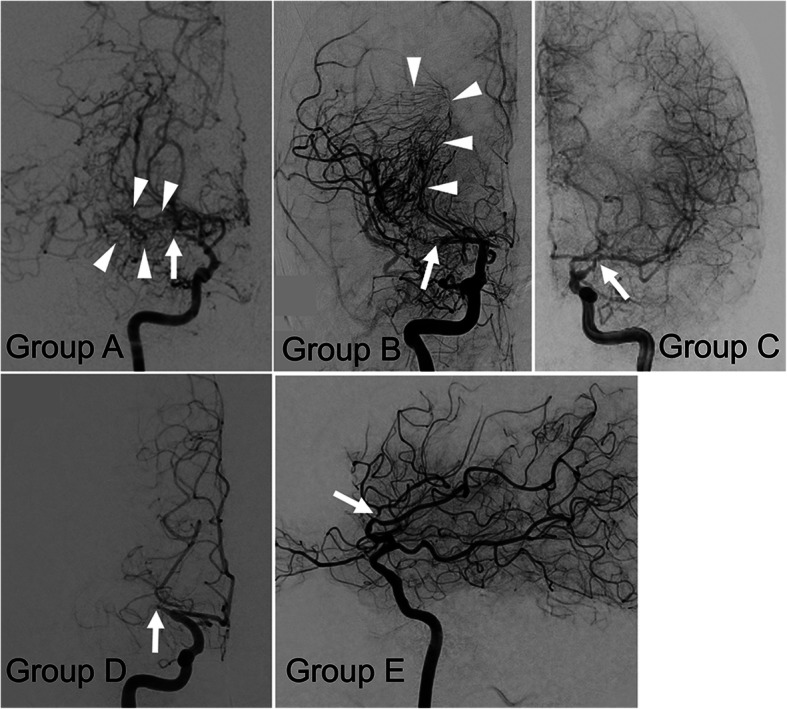
Representative DS angiograms of Groups A–E. Arrows: location of the arterial stenosis. Arrowheads: abnormal collateral vessels (moyamoya vessels)

DS angiography at diagnosis demonstrated bilateral IAS in 16 (61.5%) patients and unilateral IAS in 10 (38.5%) patients, yielding 42 affected hemispheres. Based on the site of stenosis and development of moyamoya vessels, these hemispheres were classified as follows: Group A, 4; Group B, 13; Group C, 7; Group D, 16; and Group E, 2 (Figs. 1 and 2). Further, 17 hemispheres of Groups A and B were defined as the MM type and 25 hemispheres of Groups C–E were defined as the non-MM type. In 3 patients, the MM type and non-MM type hemispheres coexisted.

Table 3 lists the characteristics of the 42 affected hemispheres. No differences were observed in terms of age and sex between the MM type and non-MM type hemispheres. Hypothyroidism was identified in 11 and 14 of the MM type and non-MM type hemispheres, respectively (*p* = 0.75). Ischemic presentation at onset was significantly more common in non-MM type hemispheres than in the MM type hemispheres (92.0% vs. 35.3%, *p* < 0.0001), whereas the hemorrhagic presentation was significantly more common in the MM type hemispheres (0% vs. 35.3%, *p* = 0.0013).

**Table 3:** Characteristics of 42 affected hemispheres

	MM type (Group A, B)	Non-MM type (Group C,D, and E)	*P* value
Number of hemispheres	17 (40.5%)	25 (59.5%)	
Mean age at diagnosis ± SD	58.4 ± 15.3	55.0 ± 11.8	0.43
Females	14 (82.4%)	18 (72.0%)	0.49
Coexisting contralateral IAS	13 (76.5%)	19 (76.0%)	0.97
Symptoms at onset			
Ischemic	6 (35.3%)	23 (92.0%)	< 0.0001
Hemorrhage	6 (35.3%)	0 (0%)	0.0013
Asymptomatic	5 (29.4%)	2 (8.0)%	0.068
Hypothyroidism	11 (64.7%)	14 (56.0%)	0.75

### Initial surgical intervention at the diagnosis of IAS

Based on the ischemic symptoms and severe hemodynamic failure, 6 (35.3%) MM type hemispheres and 3 (12.0%) non-MM type hemispheres underwent STA–MCA anastomosis within 3 months after diagnosis, whereas conservative management was initiated for the remaining hemispheres. Antiplatelet drugs were prescribed for 17 (65.4%) patients.

### IAS progression and cerebrovascular events during the follow-up periods

**Fig. 3:**
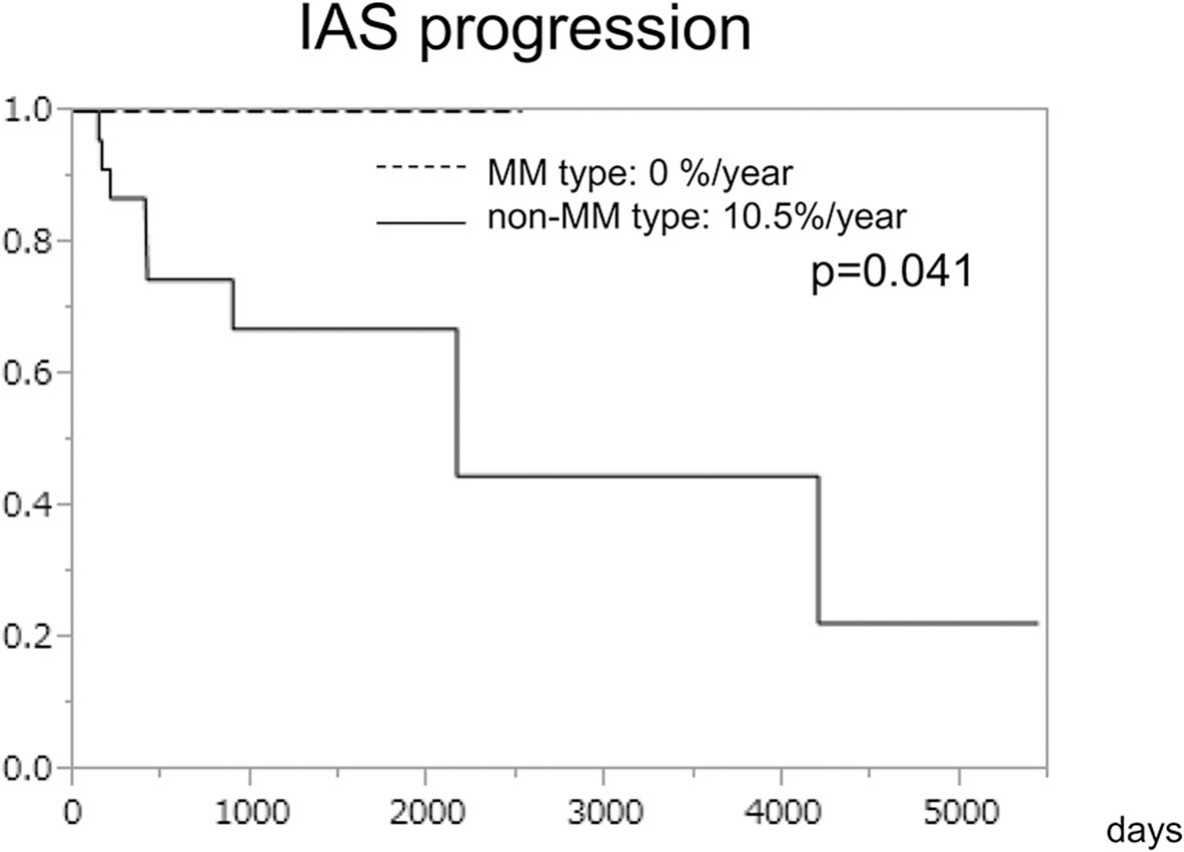
Kaplan–Meier curves for IAS progression in MM type and non-MM type hemispheres

Figure 3 shows the Kaplan–Meier curve of the MM type and non-MM type hemispheres with regard to IAS progression. During the follow-up periods of 2.5 ± 2.9 years (mean ± SD), no IAS progression was confirmed in the MM type hemispheres. However, 8 of the non-MM type hemispheres showed IAS progression at 2.9 ± 3.8 years after diagnosis (10.5%/year); this was significantly more common than that in the MM type hemispheres (*p* = 0.041). A transition from non-MM to MM or that from MM to non-MM type was not observed during the follow-up period. Multivariate analysis with calculation of hazard ratio could not be performed because the event rate was zero in the MM type hemispheres.

**Fig. 4:**
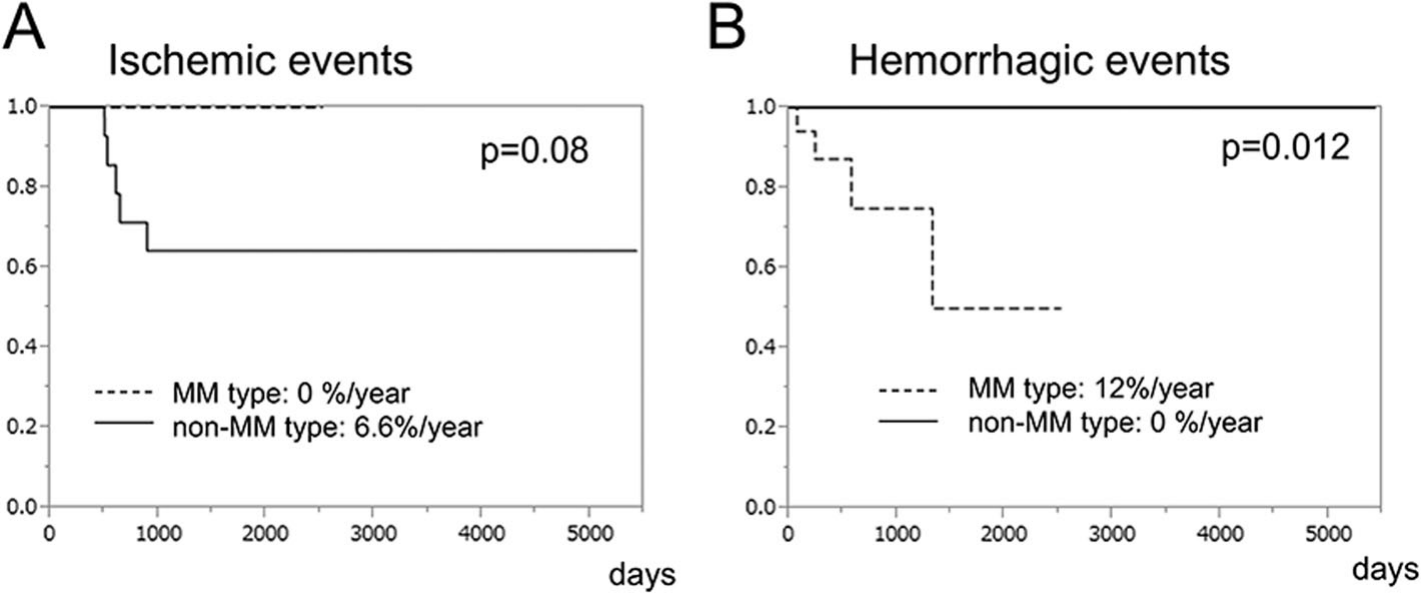
Kaplan–Meier curves for cerebrovascular events in the MM type and non-MM type hemispheres. (**a**) Ischemic and (**b**) hemorrhagic events

Figure 4 shows the Kaplan–Meier curve for ischemic (A) and hemorrhagic (B) events during the follow-up periods. Of the non-MM type hemispheres, 5 exhibited ischemic attacks (6.6%/year), whereas no ischemic event was observed in the MM type hemispheres (*p* = 0.080). Owing to ischemic events or severe hemodynamic deterioration, STA–MCA anastomosis was newly undertaken for 8 non-MM type hemispheres at 1.4 ± 0.64 y after the diagnosis. There were no additional bypass surgeries in the MM type hemispheres except for 1 case with an “initially planned” bypass that was postponed until 7 months after diagnosis because of postsurgical complications in the opposite hemisphere. Overall, surgical intervention was provided for 7 (41.2%) of the MM type hemispheres and 11 (44%) of the non-MM type hemispheres during the entire period (*p* = 0.86). Four of the MM type hemispheres had an intracerebral hemorrhage (12.0%/year), whereas none of the non-MM type hemispheres developed a hemorrhage (*p* = 0.012). Multivariate analyses for ischemic and hemorrhagic events were not conducted because the event rate was zero in the MM type for the former and in the non-MM type for the latter. Kaplan–Meier analysis showed no differences between the hemispheres with hypothyroidism and those without hypothyroidism in terms of IAS progression (*p* = 0.078), ischemic events (*p* = 0.83), and intracranial hemorrhaging (*p* = 0.39).

The mRS in the 26 patients at the time of final follow-up was 0 in 17, 1 in 5, 2 in 1, and 4 in 3 patients. Poor outcome (indicated by an mRS score of 4) was attributed to intracranial hemorrhaging in 2 of the MM type hemispheres and ischemic stroke in 1 of the non-MM type hemispheres during the follow-up period.

### Development of periventricular anastomosis in the MM type hemispheres

**Fig. 5:**
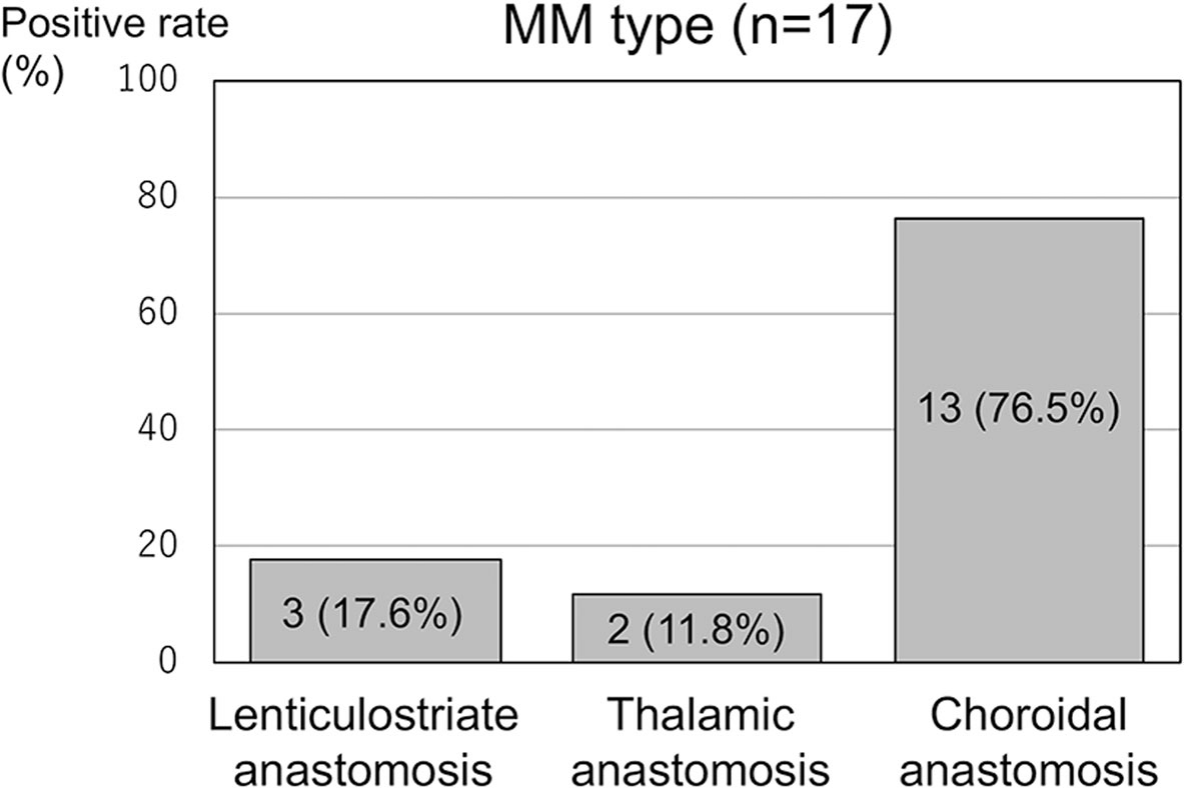
Positive rates of periventricular anastomosis in the MM type hemispheres. The rates were determined as per the criteria of the supplemental studies of the Japan Adult Moyamoya Trial

Figure 5 demonstrates the positive rate of each periventricular anastomosis in the 17 MM type hemispheres as per the criteria proposed in the supplement studies of the Japan Adult Moyamoya Trial [10, 11]. The rate of positive choroidal anastomosis, which has recently been described as a factor associated with posterior hemorrhage at a high risk of re-bleeding and a predictor of recurrent and de novo intracranial hemorrhage in moyamoya disease [10–12], was extremely high (76.5%) compared with those of lenticulostriate and thalamic anastomosis.

## Discussion

Hashimoto’s disease (chronic thyroiditis) is a common autoimmune disease that is characterized by a reduction in thyroid hormone level, thyroid goiter, and symptoms, including systemic edema, dyslipidemia, and increased body weight [1]. Chronic inflammation is attributed to the autoimmune process caused by anti-TPOAb and/or anti-TgAb [1, 2]. The prevalence is considerably high and is reportedly 46 cases/1000 [4]. In the Japanese population, anti-thyroid antibodies are reportedly present in 10.2–14.2% of women and 6–7.2% of men [13]. Several studies have recently revealed that stroke is more common in patients with Hashimoto’s disease than in the general population [3–6]. However, their intracranial vascular morphology and clinical courses have poorly been documented.

To the best of the authors’ knowledge, this is the first study to analyze detailed angiographic features and clinical courses in Hashimoto’s disease-associated IAS. A stenotic lesion was visible at the IC bifurcation, which is commonly affected in true moyamoya disease, whereas the stenosis was also observed in M1, A1, or distal ACA/MCA with an intact IC bifurcation. The affected hemispheres could be dichotomized into the MM type and non-MM type with respect to abnormal collateral vessels.

Several autoimmune disorders can reportedly induce IAS. These include Graves’ disease, systematic lupus erythematosus, and Sjögren syndrome [8, 14–17]. In Graves’ disease, which results in hyperthyroidism, recent reports have highlighted the angiographic features of IAS, demonstrating that the involvement of the IC bifurcation and moyamoya vessels can be confirmed in 79.7 and 75.9% of the cases, respectively [8]. However, in other autoimmune diseases, including Hashimoto’s disease, detailed cerebral angiographic features have not been described in the literature to date.

In the present study, the angiographic features of the non-MM type hemispheres were different from those of moyamoya disease, suggesting that this type is Hashimoto’s disease-associated vasculopathy. However, it is unclear whether the MM type is an independent entity associated with Hashimoto’s disease or it coincidentally coexists with true moyamoya disease and Hashimoto’s disease. This question arises because the prevalence of Hashimoto’s disease is considerably high in the population [1, 18, 19]. This should be considered particularly for Group A, consistent with the diagnostic criteria of moyamoya disease. However, Group B that accounted for 76% of the MM type hemispheres, lacked stenosis of the IC bifurcation, indicating that Hashimoto’s disease-associated MM type can exist as an independent entity.

It is noteworthy that the non-MM type hemispheres were at a significantly higher risk of stenosis progression than the MM type hemispheres. Furthermore, ischemic events were observed only in the non-MM type hemispheres during the follow-up periods. In the present study, bypass surgery was performed for 4 non-MM type hemispheres during the follow-up period because of asymptomatic stenosis progression. This suggested that the ischemic risk would have been higher if surgery was not performed. In the Kaplan–Meier curve analysis for ischemic events, the additional bypass surgeries were not considered (Fig. 4a). The result was comparable when surgical intervention was considered as “censoring” (*p* = 0.084).

Further, it is noteworthy that MM type hemispheres had a high risk of intracranial hemorrhage. Hemorrhage at the initial presentation was observed only in MM type hemispheres. Furthermore, only MM type hemispheres developed hemorrhaging during the follow-up periods with an annual rate of 12.0%/year. The reported annual re-bleeding rate of hemorrhagic moyamoya disease is 7.1–7.6%, and recent studies have highlighted the importance of periventricular anastomosis, particularly on choroidal anastomosis as a risk factor of recurrent and de novo intracranial hemorrhage in moyamoya disease [10–12, 20–22]. In the present study, 76.5% of the MM type hemispheres exhibited positive choroidal anastomosis. This may explain the high hemorrhagic rate in MM type hemispheres in Hashimoto’s disease-associated IAS.

The mechanism of IAS in Hashimoto’s disease has been poorly documented by previous studies. In Graves’ disease, a T-cell-mediated immunological mechanism and/or sympathetic-nerve-mediated vasculopathy are presumed to cause IAS [3, 23, 24]. An elevated cerebral metabolism of oxygen and hypercoagulability caused by elevated thyroid hormones (hyperthyroidism) has the ability to aggravate cerebral ischemia [3, 23, 24]. However, for Hashimoto’s disease, no promising hypothesis explaining the progression of vascular stenosis has emerged. Recent reports have documented a high rate of positive anti-thyroid antibodies in moyamoya disease [5, 23]. Kim SJ et al. compared the positive rate of antimicrosomal antibodies (AMA) that is equivalent to TPOAb among the following three groups: moyamoya disease, non-moyamoya stroke, and normal controls [5]. The prevalence of AMA was significantly higher in the presence of moyamoya disease. Moreover, Tashiro et al. have examined the association between human leukocyte antigen (HLA) and moyamoya disease [7]. They showed that HLA-DRB1*04:10 was a risk allele for moyamoya disease and that thyroid diseases, such as Graves’ and Hashimoto’s disease, were more common in HLA-DRB1*04:10-positive patients with moyamoya disease than in HLA-DRB1*04:10-negative patients. These data suggested a potential immunological mechanism in Hashimoto’s disease-associated IAS. Hypothyroidism itself reportedly increases the risk for cardiovascular events and atherosclerosis [25, 26]. However, in the present study, decreased thyroid hormone levels were not associated with cerebrovascular events or IAS progression. Further studies are required to elucidate the pathophysiology of Hashimoto’s disease-associated IAS.

There are several limitations of the present study. First, this was a retrospective cohort study with a relatively small sample size, and selection bias could not be eliminated because screening for anti-thyroid antibodies was not performed for all patients with IAS. Second, the follow-up periods were insufficient. Third, analyses of the subsequent vascular events were not based on a purely natural course because some patients underwent bypass surgery at the beginning of the follow-up period. Despite the limitations, this study showed the outcomes under a certain management strategy, including surgical intervention for the hemodynamically impaired patients.

## Conclusions

Hashimoto’s disease-associated IAS exhibits several types of angiographic morphologies. Non-MM type hemispheres are at risk of IAS progression and ischemic events during the short term, whereas MM type hemispheres can develop subsequent intracranial hemorrhaging at a high rate. Although the pathophysiology of Hashimoto’s disease-associated IAS requires further elucidation, these results strongly suggest the importance of screening for anti-thyroid antibodies and careful management based on vascular morphology in adult patients with IAS.

## Data Availability

The datasets generated and/or analyzed during the current study are not publicly available due to personal information included, but are available from the corresponding author on reasonable request.
